# Quality of life and its association with direct medical costs for COPD in urban China

**DOI:** 10.1186/s12955-015-0241-5

**Published:** 2015-05-14

**Authors:** Minmin Wu, Qi Zhao, Yue Chen, Chaowei Fu, Biao Xu

**Affiliations:** School of Public Health and Key Laboratory of Public Health Safety, Fudan University, Shanghai, 200032 China; Faculty of Medicine, University of Ottawa, Ottawa, ON Canada

**Keywords:** Chronic obstructive pulmonary disease, Quality of life, A cross-sectional study, Medical cost

## Abstract

**Background:**

Chronic obstructive pulmonary disease (COPD) is one of the leading causes of death worldwide. Few studies have focused on the quality of life (QoL) associated medical costs for COPD in China.

**Methods:**

A cross-sectional survey of 678 COPD patients was conducted in four major cities (Beijing, Shanghai, Guangzhou and Chengdu), China, in 2011. Data on socio-demographic information, health conditions and medical costs were collected through a face-to-face interview combined with medical record searching. The EuroQol (EQ-5D) health questionnaire was applied to assess the QoL of COPD patients.

**Results:**

Among 678 patients with COPD, nearly 40% had difficulties in mobility, usual activities and pain/discomfort, one third had various degrees of anxiety/depression, and one fifth had difficulties in self-care. The COPD patients had a median utility score of 0.768 and a median visual analog scale score of 70. The degree of difficulties in any dimensions significantly increased, and utility and health scores decreased with severity of the disease. Age, gender and disease severity were significantly associated with the quality of life after taking other covariates into consideration. Poorer QoL was a significant indicator of higher direct medical costs for COPD patients.

**Conclusion:**

Impaired quality of life was significantly linked to increased medical costs for COPD patients and could be an important measure for policy- and decision-making in COPD care.

## Background

Chronic obstructive pulmonary disease (COPD) is ranked the fourth of the 10 leading causes of death, and killed approximately 3 million people (5.8% of all deaths) globally in 2011 [1]. Compared to a decrease in cardiovascular mortality and stabilization of cancer mortality worldwide, mortality from COPD is increasing in European countries [2]. In China, the prevalence of COPD was as high as 8.2% in 2008 for adults aged 40 years or above based on data from a national survey [3].

COPD is a well-recognized high economic burden disease. In 2010, the costs of COPD were projected to be approximately US$50 billion, which included $20 billion in indirect costs and $30 billion indirect health care expenditures in the U.S. [4]. How to reduce such enormous costs for COPD care is an unmet challenge to all countries.

COPD is characterized by airflow limitation that is not fully reversible. It is also an irreversible respiratory disease that can seriously reduce people’s physical capacity, social functioning and subjective well-being. In order to assess how COPD patients themselves perceive their lives, various forms of instruments are used, most often based on self-report [5]. The EuroQol (EQ-5D) is one instrument to measure Health related quality of life (HRQoL) that has been calibrated with different populations, and has been used widely. It is brief and simple, and patients can easily understand and complete. The EQ-5D is also easy to score and interpret. Assessments of HRQoL using generic instruments like the EQ-5D allow for comparisons with other chronic diseases as well as comparisons with healthy populations to estimate the incremental burden of COPD [6].

HRQoL is a common measure used in the evaluation of healthcare interventions. A number of studies found that proper health interventions could both improve the quality of life (QoL) and reduce the medical costs of COPD patients [6-8]. QoL is likely an important predictor for subsequent COPD exacerbation, hospitalization and mortality, and is related to the severity of COPD, which all are measures associated with a high healthcare expenditure [9-12]. Also, QoL is strongly associated with investment beliefs and their behaviors including healthcare seeking [13-16]. Therefore, QoL may be a better predictor for medical costs than others, and can be used for policy- and decision-making. So far, there has been limited information on the QoL for COPD patients in China and the relationship between QoL and direct medical costs has not been clear. This study intended to understand the QoL of COPD patients and the relationship to medical direct costs in China.

## Materials and methods

### Study site and subjects

A cross-sectional study was carried out in Beijing, Guangzhou, Shanghai and Chengdu from March to June 2011. Fifteen community health centers (or hospitals) were involved to recruit subjects from the communities. Patient inclusion criteria included that a patient: 1) Met the diagnosis criterion of COPD provided by the Chinese Society of Respiratory Diseases in 2007 [17]; 2) Received the assessment of lung function at least once in the past 12 months; 3) was able to provide informed consent and medical chart; 4) was able to complete the questionnaire interview; and 5) was a local resident who lived in the city for at least 2 years. Patients who had any unstable comorbidities or any severe conditions needed to be treated as inpatients were excluded from the study. All subjects had lung function testing during the survey. Totally, 678 out of 721 eligible COPD patients were included in this study, with a response rate of 94%.

### Questionnaire

The EQ-5D health questionnaire was applied to assess the QoL for COPD patients [7,9]. The self-classifier consists of five dimensions (mobility, self-care, usual activities, pain/discomfort, and anxiety/depression), each with three levels of response (level 1 = no problems, level 2 = some problems, or level 3 = inability or extreme problems). The utility score is typically interpreted along a continuum where 1 represents best possible health and 0 represents dead, with some health states considered worse than dead. In addition to the self-classifier, the EQ-5D contains a 20 cm visual analog scale (VAS) ranging from 0 (worst imaginable health) to 100 (best imaginable health) along which the respondent rates their health today. The index-based utility scores can be used to compare a burden of disease across different conditions and facilitate the calculation of quality adjusted life years (QALYs) that are incorporated into economic evaluations of health care interventions [8]. A single summary score was generated by applying societal preference weights to a health state classifier completed by patients.

### Data collection and quality control

Data collectors were health workers who were recruited from each study site and trained by academic investigators. The study subjects were face-to-face interviewed with a structured questionnaire, and their medical charts were also reviewed and the details of medical care utilization were obtained. The data of medical costs were collected from their medical charts and bill payments.

All questionnaires were double-checked while they were filled out. An interview was conducted by two investigators - one was in charge of the interview and another had a double-check and made sure that the interview information was recorded correctly, as one measure of quality control. Fifteen subjects were randomly sampled in each city and a total sixty subjects were re-interviewed by the telephone after 2–3 months to check the reproducibility of the questionnaire information for quality control. The key variables included sex, smoking status, disease severity, number of outpatient visits and hospital admission information. The reliability was satisfactory with Kappa values of 0.93 - 1.00 for key categorical variables and intraclass correlation coefficients of 0.96 - 0.98 for key continuous variables.

### Informed consent

A written informed consent was obtained from all subjects. They were free to withdraw from the study at any time with no negative consequences. The ethic approval for this study was issued by the Institutional Review Board of the Fudan University School of Public Health.

### Data analysis

Subjects were grouped into four categories of severity (mild, moderate, severe and very severe) for COPD based on the criteria established by the Global Initiative for Chronic Obstructive Lung Disease (GOLD) [18]. Annual direct medical costs for each patient were calculated based on the total expenditures in the past 12 months of this interview and records for treating COPD, including co-payments, diagnoses, treatments, diagnostic tests, prescription drugs, medical supplies, and use of oxygen in both outpatient visits and hospital admissions.

All the data were double input into a database established through EpiData3.1 Chinese version. Statistical analysis was completed by using SPSS16. Chi-square test was used for testing the differences of five dimensions, and Kruskal-Wallis test for comparing the utility scores and VAS scores. The estimation of EQ-5D utility was based on the Japanese population index set [19]. The annual direct medical costs and the QoL of EQ-5D utilities were analysed by comparing the top 25th percentile with the low 75th percentile. In logistic regression analysis, the associations of factors with healthcare costs and QoL were assessed by calculating crude and adjusted odds ratios (OR) and 95% confidential intervals (CI). Spearman’s correlations were calculated for the relationships among QoL and annual direct medical costs. An alpha level of ≤0.05 (two sides) was considered to be statistically significant.

## Results

### The general and clinical characteristics

Of the 678 participants, 72.9% were male. A majority of them were retired with an average age of 70.4 ± 10.1 years. Three quarters (74.6%) of them had received 9 years of education or more. Of them, 19.4% were current smokers, and almost all of them (95.7%) had at least one type of medical insurances. The median age at the diagnosis of COPD was 63.9 years (means: 61.6 ± 13.7 years) and the median duration of COPD was 3.8 years (means: 8.8 ± 11.6 years). About one quarter had a family history of chronic lung diseases, and most of them (79%) had at least one co-morbid chronic disease. The median forced expiratory volume in one second (FEV_1_) was 1.3 liters (mean: 1.6 ± 1.3 liters) and the median FEV_1_% predicted was 53.0 (mean: 54.5 ± 23.0). According to the criteria of GOLD, 20.6% were in mild stage, 43.7% in moderate stage, 26.7% in severe stage, and 9.0% in very severe stage. (Table 1).

**Table 1:** **Demographic and clinical characteristics of COPD patients**

**Characteristics**	**Beijing (%)**	**Guangzhou (%)**	**Shanghai (%)**	**Chengdu (%)**	**Total (%)**
Age (years)*	73.3 (71.6 ± 10.3)	68.8 (68.0 ± 8.6)	69.6 (68.8 ± 11.1)	74.2 (73.3 ± 9.4)	71.8 (70.4 ± 10.1)
Gender (Male)	100 (58.8)	152 (86.9)	125 (74.4)	117 (70.9)	494 (72.9)
Education (<9 years)	53 (31.4)	37 (21.1)	36 (21.4)	53 (32.1)	179 (26.4)
Medical insurance	166 (97.6)	156 (89.1)	164 (97.6)	163 (98.8)	649 (95.7)
Smoking	40 (23.7)	40 (22.9)	25 (14.9)	26 (15.8)	131 (19.4)
Age at diagnosis (years)*	64.0 (61.4 ± 13.9)	66.5 (64.7 ± 9.7)	61.9 (61.3 ± 13.3)	60.4 (59.4 ± 15.5)	63.9 (61.7 ± 13.3)
Course of illness (years)*	6.6 (10.2 ± 12.2)	1.4 (3.2 ± 4.6)	3.8 (7.6 ± 9.4)	8.6 (13.9 ± 14.2)	3.8 (8.7 ± 11.3)
Family history	39 (23.1)	40 (22.9)	38 (22.6)	61 (37.2)	178 (26.3)
FEV1 (liter)*	1.5 (1.4 ± 0.5)	1.4 (1.5 ± 1.0)	1.2 (2.2 ± 2.2)	1.1 (1.2 ± 0.7)	1.3 (1.6 ± 1.3)
FEV1% predicted*	58.7 (57.4 ± 19.5)	56.0 (57.8 ± 24.6)	49.0 (52.1 ± 22.4)	47.0 (49.9 ± 24.4)	53.0 (54.5 ± 23.0)
Co-morbidities	158 (92.9)	96 (54.9)	130 (77.4)	150 (91.5)	534 (78.9)
Current severity level					
Mild	31 (18.2)	35 (20.0)	37 (22.0)	37 (22.4)	140 (20.6)
Moderate	86 (50.6)	79 (45.1)	61 (36.3)	70 (42.4)	296 (43.7)
Severe	40 (23.5)	39 (22.3)	57 (33.9)	45 (27.3)	181 (26.7)
Very severe	13 (7.6)	22 (12.6)	13 (7.7)	13 (7.9)	61 (9.0)
Total	170 (100)	175 (100)	168 (100)	165 (100)	678 (100)

*Median (Mean ± standard deviation).

Abbreviations: *FEV1* forced expiratory volume in one second, *FEV1% predicted* the percentiles of forced expiratory volume in one second to the predicted value.

### QoL and related factors

Based on information collected using the EQ-5D questionnaire, nearly 40% of COPD subjects had difficulties in mobility, usual activities and pain/discomfort, one third had anxiety/depression and one fifth had difficulties in self-care (Table 2). The median of utility score was 0.768 and the median of VAS score was 70. The degree of difficulties significantly increased with the severity of COPD while the utility and health scores decreased. Logistic regression analysis showed that age and gender, severity of COPD were significantly associated with the quality of life for COPD patients after adjusting for household income, having a medical insurance, duration of COPD, presence of a comorbid condition and study site (Table 3).

**Table 2:** **Distribution of quality of life measure for COPD patients by current severity levels**

**Current severity**	Mobility (%)*			Self-care (%)*			Usual activities (%)*			Pain/Discomfort (%)*			Anxiety/depression (%)*			VAS score**	Utilities score**
	**1**	**2**	**3**	**1**	**2**	**3**	**1**	**2**	**3**	**1**	**2**	**3**	**1**	**2**	**3**		
Mild	80.6	19.4	0.0	93.5	6.5	0.0	86.3	12.9	0.7	76.3	23.0	0.7	72.7	26.6	0.7	70.0 (71.8 ± 13.3)	0.848 (0.786 ± 0.085)
Moderate	67.6	29.1	3.4	85.8	11.1	3.0	67.6	27.4	5.1	62.8	35.1	2.0	73.0	24.7	2.4	70.0 (67.1 ± 16.7)	0.773 (0.734 ± 0.158)
Severe	44.8	52.5	2.8	75.1	22.1	2.8	44.8	48.1	7.2	54.7	42.5	2.8	67.4	30.4	2.2	67.0 (63.5 ± 16.3)	0.724 (0.691 ± 0.155)
Very severe	31.1	65.6	3.3	65.6	29.5	4.9	32.8	59.0	8.2	47.5	47.5	4.9	63.9	31.1	4.9	60.0 (61.3 ± 15.8)	0.675 (0.655 ± 0.151)
p value	<0.001			0.016			<0.001			<0.001			<0.001			<0.001	<0.001
Total	60.9	36.6	2.5	82.7	14.8	2.5	62.2	32.8	5.0	62.0	35.7	2.2	70.6	27.2	2.2	70.0(66.6 ± 16.2)	0.768 (0.726 ± 0.150)

*1 = no problem; 2 = moderate or some problem; 3 = inability or extreme problem.

**Median (Mean ± standard deviation).

Abbreviation: *VAS* visual analog scale.

**Table 3:** **Factors associated with utilities of EQ-5D in COPD patients**

**Variables**	**Top 25%**	**Low 75%**	**cOR (95% CI)**	**p value**	**aOR (95% CI)** ^*^	**p value** ^*^
**Age (years)**	-	-	0.96 (0.95,0.98)	<0.001	0.98 (0.96,0.99)	0.012
**Gender**						
Male	175	318	1.48 (1.02,2.14)	0.041	1.65 (1.06,2.58)	0.028
Female	50	134	1.00	-	1.00	-
**Current severity**						
Mild	72	67	12.04 (4.55,31.86)	<0.001	14.76 (5.33,40.89)	<0.001
Moderate	108	188	6.43 (2.50,16.55)	<0.001	7.15 (2.69,18.97)	<0.001
Severe	40	141	3.18 (1.19,8.46)	0.021	3.43 (1.25,9.38)	0.016
Very severe	5	56	1.00	-	1.00	-
**Total**	225	452	-	-	-	-

*Variables included in the logistic regression model: study site, average household income, having medical insurance(s), duration of COPD and presence of comorbidities.

Abbreviations: *aOR* adjusted odds ratio, *cOR* crude odds ratio, *CI* confidence interval, *COPD* chronic obstructive pulmonary disease.

### QoL and annual direct medical costs

As shown in Table 4, the QoL of patients was inversely related to the annual direct medical costs for COPD care.. After adjustment for study site, age, gender, household income, severity and duration of COPD, having a medical insurance and having a co-morbid condition, the association was less strong but remained significant. Further, annual direct medical costs were negatively associated with both VAS scores (r = −0.198, p < 0.001) and utility scores (r = −0.336, p < 0.001), and also statistically associated with five dimensions including mobility (r = 0.347, p < 0.001), self-care (r = 0.310, p < 0.001) usual activities (r = 0.376, p < 0.001), pain/discomfort (r = 0.166, p < 0.001) and anxiety/depression (r = 0.106, p = 0.006).

**Table 4:** **The relationship between direct medical costs for COPD care and quality of life of patients**

**Utilities of EQ5D**	**Annual direct medical cost**		**cOR (95% CI)**	**p value**	aOR (95% CI)*	p value*
	**Top 25%**	**Lower 75%**				
Lower 75%	139	305	3.28	<0.001	2.10	0.003
			(2.09,5.13)		(1.29,3.44)	
Top 25%	27	194	1.00	-	1.00	-

*Variables included in the logistic regression model: study site, age, gender, household income, severity and duration of COPD, having medical insurance(s) and presence of comorbidities.

Abbreviations: *aOR* adjusted odds ratio, *cOR* crude odds ratio, *CI* confidence interval, *COPD* chronic obstructive pulmonary disease.

## Discussion

### Impaired QoL for COPD patients

COPD is significantly associated with lower QoL, and QoL has been recognized as an important outcome for COPD. Patient reported outcomes such as health status and quality of life are accepted as routine measures in clinical studies, particularly in respiratory-related chronic conditions [20-22]. In this study, we observed impaired quality of life for COPD patients living in urban China using the EQ-5D, and the utility and VAS scores declined with the severity of COPD, which were similar to the results from the UK and the U.S. according to the utility and VAS scores [10,23,24].

### QoL in COPD and possible related factors

Age and gender and severity of COPD were independent factors significantly associated with the quality of life for COPD patients after adjusting for covariates. In urban China, younger and male patients had higher proportions to have better utility scores, which was similar to the results from previous studies [4,20,25,26]. This result suggested that more attentions should be paid to older and female COPD patients when developing interventions to improve their QoL.

In addition, as shown in Tables 2 and 3, the severity of COPD was significantly related to the EQ-5D utility scores, VAS scores and each dimension of EQ-5D. It is expected that the QoL in COPD patients decreased with disease severity as reported in the previous studies [23,27-29]. The distinction in utility scores was relatively small between the GOLD stages I and II groups, which was also similar to previous observations [30]. The classification of GOLD stage is based on the levels of forced expiratory volume in one second, which are only weakly correlated with the EQ-5D utility scores [24,31]. The limited ability to distinguish between milder stages of disease may be a limitation in the discriminative ability of the EQ-5D compared to some disease-specific instruments like the St. George’s Respiratory Questionnaire [24,25,32].

### QoL and direct medical costs for COPD

In this study, it was observed that patients with poorer QoL had higher direct medical costs for COPD care than those with better QoL, suggesting that to improve QoL may help reduce direct medical costs for COPD patients. QoL is likely an independent and important measure for subsequent COPD exacerbation, hospitalization, and mortality, and disease severity is associated with a high healthcare expenditure [10-12]. Further, all five dimensions of EQ-5D had positive correlations to annual direct medical costs, especially for two dimensions of mobility and usual activities. The dimension of anxiety/depression had a relatively low correlation coefficient, As an important part of QoL, anxiety or depressive moods are strongly associated with investment beliefs. They have been shown to be a primary predictor of physically active behavior, and physically active behavior can help improve HRQoL and save COPD medication costs [13-16]. The link between QoL and medical costs due to COPD informs health care professionals and health policy makers changes in QoL affecting direct medical costs and helps in designing interventions to improve the QoL of COPD patients.

The strengths and limitations of our study should be noted. This was a hospital-based cross-sectional study in urban China. This study carried out in four metropolitan areas, with a high response rate. Quality control was carefully planned and conducted. The QoL could well reflect the current status of Chinese COPD patients living in major urban areas. An inverse relationship between QoL of patients and direct medical costs for COPD care had not been previously studied in China. Although this is a cross-sectional study and the evidence is weak for possible causal association, it is not likely that a higher cost for COPD healthcare would result in lower QoL for COPD patients. Another limitation of the study is that a random sampling method was not used, and therefore, a caution is needed for a generalization to broader population. Also, in this study, there may be a recall bias for self-reporting information. Some information was confirmed by checking their medical records. The estimation of the EQ-5D utility was based on the Japanese population index set since a Chinese population index set was unavailable. Among three index sets of the EQ-5D utility most frequently used, the Japanese one as compared with American and British ones seemed to more appropriate for Chinese population.

## Conclusion

We measured impaired quality of life for COPD patients living in urban China using the EQ-5D. Age, gender and severity stages of COPD were associated with patients’ QoL. The QoL of patients had a strong negative correlation with direct medical costs for COPD care, which suggests that QoL can be an important measure for COPD care, and improving QoL would reduce direct medical costs.
